# NADPH Oxidase 4 Mediates Insulin-Stimulated HIF-1α and VEGF Expression, and Angiogenesis *In Vitro*

**DOI:** 10.1371/journal.pone.0048393

**Published:** 2012-10-29

**Authors:** Dan Meng, Aihong Mei, Junxu Liu, Xueling Kang, Xianglin Shi, Ruizhe Qian, Sifeng Chen

**Affiliations:** 1 Department of Physiology and Pathophysiology, Fudan University Shanghai Medical College, Shanghai, China; 2 Department of Diagnosis, Shanghai Tenth People's Hospital, Tongji University School of Medicine, Shanghai, China; 3 Graduate Center for Toxicology, University of Kentucky, Lexington, Kentucky, United States of America; University of Illinois at Chicago, United States of America

## Abstract

Acute intensive insulin therapy causes a transient worsening of diabetic retinopathy in type 1 diabetes patients and is related to VEGF expression. Reactive oxygen species (ROS) have been shown to be involved in HIF-1α and VEGF expression induced by insulin, but the role of specific ROS sources has not been fully elucidated. In this study we examined the role of NADPH oxidase subunit 4 (Nox4) in insulin-stimulated HIF-1α and VEGF expression, and angiogenic responses in human microvascular endothelial cells (HMVECs). Here we demonstrate that knockdown of Nox4 by siRNA reduced insulin-stimulated ROS generation, the tyrosine phosphorylation of IR-β and IRS-1, but did not change the serine phosphorylation of IRS-1. Nox4 gene silencing had a much greater inhibitory effect on insulin-induced AKT activation than ERK1/2 activation, whereas it had little effect on the expression of the phosphatases such as MKP-1 and SHIP. Inhibition of Nox4 expression inhibited the transcriptional activity of VEGF through HIF-1. Overexpression of wild-type Nox4 was sufficient to increase VEGF transcriptional activity, and further enhanced insulin-stimulated the activation of VEGF. Downregulation of Nox4 expression decreased insulin-stimulated mRNA and protein expression of HIF-1α, but did not change the rate of HIF-1α degradation. Inhibition of Nox4 impaired insulin-stimulated VEGF expression, cell migration, cell proliferation, and tube formation in HMVECs. Our data indicate that Nox4-derived ROS are essential for HIF-1α-dependent VEGF expression, and angiogenesis *in vitro* induced by insulin. Nox4 may be an attractive therapeutic target for diabetic retinopathy caused by intensive insulin treatment.

## Introduction

Acute intensive insulin therapy causes a transient worsening of diabetic retinopathy with neovascularization in type 1 diabetes patients and is related to VEGF expression [1]. Insulin-induced VEGF expression has been reported to be mediated through the activation of PI 3-kinase/AKT and p44/42 MAPK pathways, and hypoxia-inducible factor-1α (HIF-1α) [2]. Insulin has been shown to increase intracellular reactive oxygen species (ROS) production in many cell types such as HepG2 cells [3], 3T3L1 adipocytes [4], human fibroblasts [5], and retinal microvascular endothelial cells [6]. ROS produced by insulin are involved in HIF-1α and VEGF expression [3], [7]. However, the specific ROS sources that mediate insulin-induced HIF-1α and VEGF expression are not completely defined.

ROS such as superoxide, hydrogen peroxide, and hydroxyl radical are involved in the signaling pathways that mediate many stress and growth responses, including angiogenesis [8]–[10]. Nox-family NADPH oxidases have proven to be a major source of ROS production in various cell types and have crucial roles in various physiological and pathological processes [11]–[14]. We showed previously that NADPH oxidase subunit 4 (Nox4)-derived ROS generation in response to insulin-like growth factor-1 (IGF-I) was linked to cell proliferation and migration in vascular smooth muscle cells (VSMCs) [15]–[16]. There is evidence that the level of Nox4 expression in endothelial cells *in vivo* is high as compared with other Nox isoforms [17]. Nox4 is important for ROS generation in human endothelial cells [18]. Moreover, Nox4 mediates endothelial responses to angiotensin-II [19], transforming growth factor-β [20] and epidermal growth factor [21], which include migration and proliferation. These data suggest that Nox4 has a critical role in modulating endothelial cell physiology.

In this study we examined the role of Nox4 in insulin-stimulated HIF-1α and VEGF expression, and angiogenic responses in human microvascular endothelial cells (HMVECs). Here we demonstrated that Nox4 mediates insulin-stimulated ROS generation, and angiogenesis *in vitro*, Nox4-derived ROS are essential for HIF-1α-dependent VEGF expression induced by insulin.

**Figure 1:**
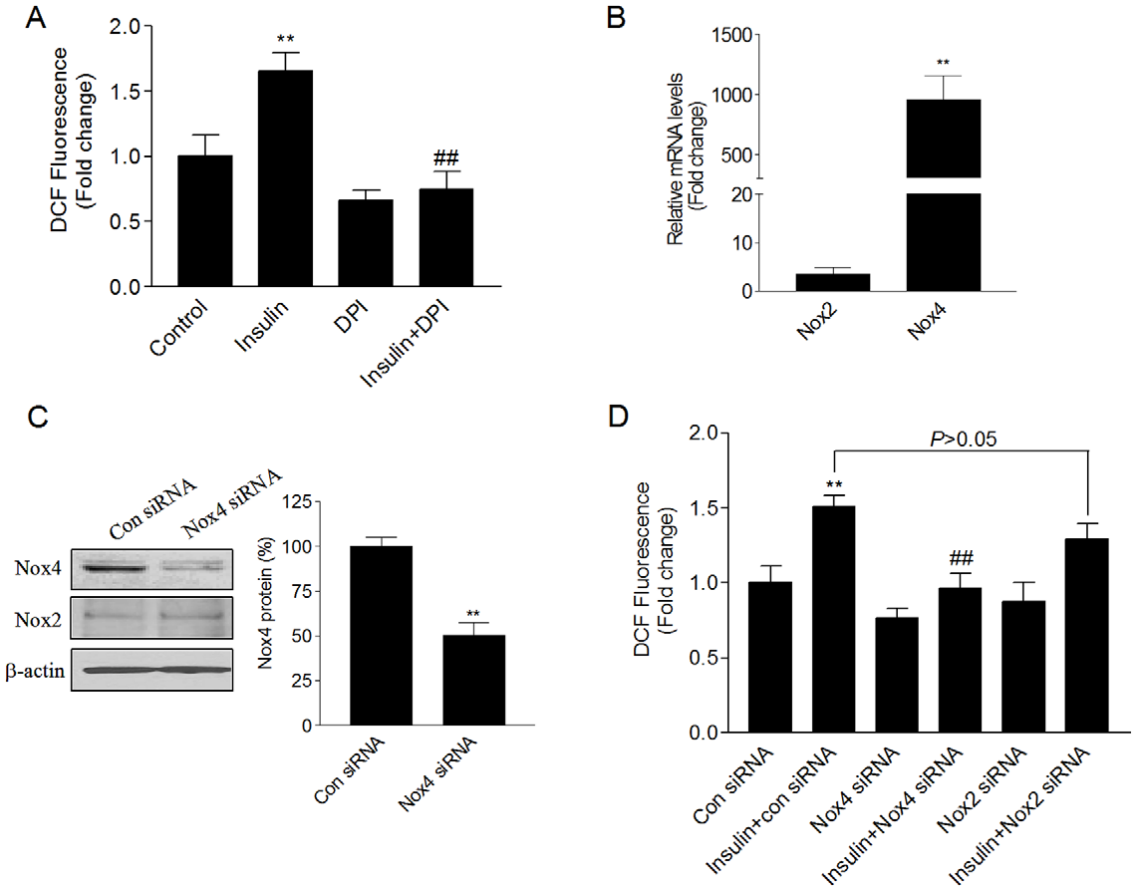
Insulin increases ROS production through a Nox4-based NADPH oxidase in HMVECs. (A) Growth-arrested HMVECs were pre-incubated with DPI (5 μM) for 30 minutes, and then stimulated with insulin (100 nM) for 5 minutes. Measurement of ROS formation by DCF-DA method was as described in Methods. (B) mRNA expression of Nox2 and Nox4 in HMVECs was determined by real-time RT-PCR. (C) HMVECs were transfected with scrambled (control) or Nox4 siRNA. In 48 hours, the cells were harvested for detecting the Nox4 and Nox2 protein. (D) HMVECs were transfected with control siRNA, Nox4 siRNA, or Nox2 siRNA. In 24 hours the cells were growth-arrested, followed by stimulation with insulin (100 nM) for 5 minutes. ROS production was determined using DCF-DA as substrate. **P<0.01 vs. the control; ##*P*<0.01 vs. the insulin-treated group.

## Materials and Methods

### Materials

Recombinant human insulin, diphenylene iodonium (DPI), cyclohexamide (CHX), and antibody against β-actin were purchased from Sigma-Aldrich (St. Louis, MO). 2′,7′-dichlorodihydrofluorescein diacetate (DCF-DA) was from Molecular Probes (Eugene, OR). Antibodies against phospho-insulin receptor β-subunit (Tyr 1162/1163), insulin receptor β-subunit, phospho-IRS-1(Tyr 1229), IRS-1, phospho-ERK1/2, ERK2, Nox4, Nox2, MKP-1, SHIP-1, and human Nox4, Nox2 siRNA were from Santa Cruz Biotechnology (Santa Cruz, CA). Antibodies against phospho-AKT and AKT were from Cell Signaling (Beverly, MA). Antibody against HIF-1α and Matrigel were purchased from BD Pharmingen (San Diego, CA). For the detailed antibodies information please see Table S1.

**Figure 2:**
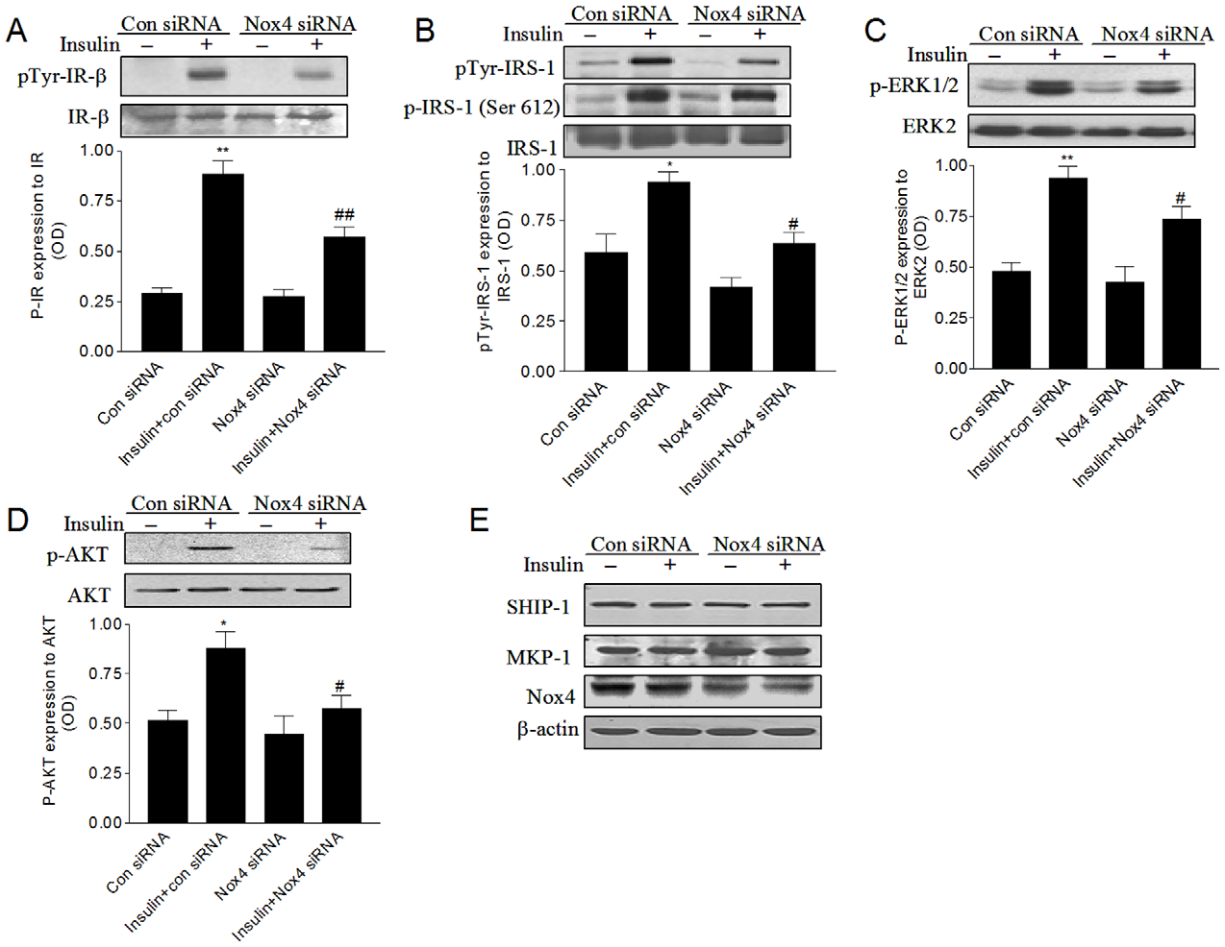
Nox4 is involved in insulin-stimulated insulin receptor and IRS tyrosine phosphorylation, ERK1/2, and AKT activation. HMVECs were treated with Nox4 siRNA or scrambled control siRNA for 24 hours, and then incubated with serum-free medium for 16 hours. (A) The tyrosine phosphorylated and total insulin receptor, (B) The tyrosine phosphorylated, the serine phosphorylated, and total IRS-1 protein levels were measured after stimulation with 100 nM insulin for 5 minutes. (C) Phosphorylated and total ERK1/2, (D) phosphorylated and total AKT, and (E) SHIP-1, MKP-1, Nox4, and β-actin protein expressions were determined after stimulation with 100 nM insulin for 30 minutes. Densitometry values (bottom) were normalized as the indicated. *P<0.05, **P<0.01 vs. the control siRNA group; #*P*<0.05, ##*P*<0.01 vs. the insulin+control siRNA group.

### Cell culture

Human microvascular endothelial cells (HMVECs) immortalized with the human telomerase catalytic protein (hTERT) [22] were a kind gift from Dr. Rong Shao (University of Massachusetts, Springfield, MA). The cells were cultured as described previously [22].

**Figure 3:**
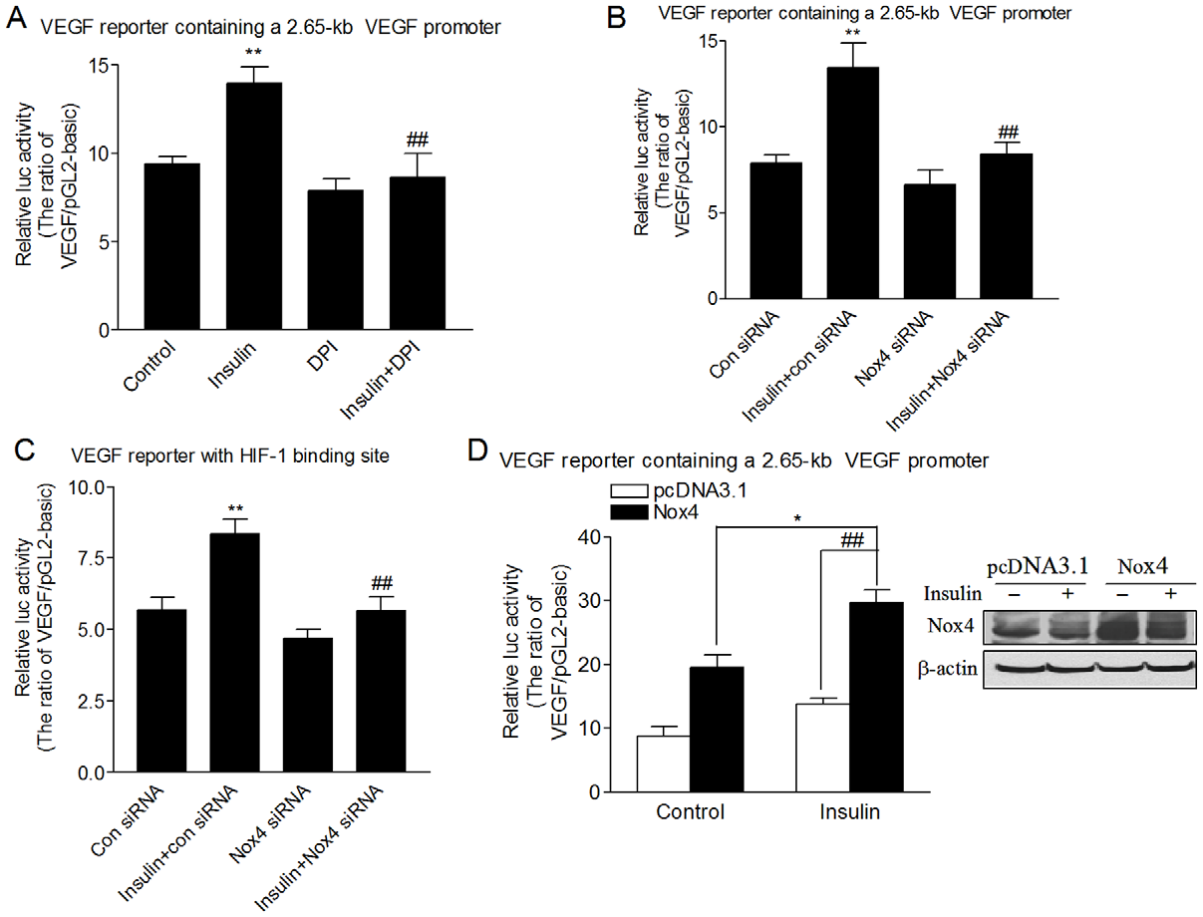
Nox4 mediates insulin-induced VEGF transcriptional activation through HIF-1. (A) HMVECs were co-transfected with a VEGF reporter containing a 2.65-kb human VEGF promoter fragment or pGL2-basic luciferase reporter and β-gal plasmid. In 24 hours the cells were growth-arrested, followed by stimulation with insulin (100 nM) for 24 hours in the presence or absence of DPI (5 μM). (B) VEGF reporter (2.65-kb) or (C) VEGF reporter containing the HIF-1 binding site (46-bp) and β-gal plasmids were cotransfected with the control siRNA or Nox4 siRNA. In 24 hours, the cells were growth-arrested and then treated with insulin (100 nM) for 24 hours. (D) VEGF reporter (2.65-kb) and β-gal plasmids were cotransfected with Nox4 or pcDNA3.1 plasmid. In 24 hours, the cells were growth-arrested and then treated with insulin (100 nM) for 24 hours. Relative luciferase activity was determined by the ratio of luciferase/β-galactosidase activity, and expressed as the ratio of VEGF/pGL2-basic. **P<0.01 vs. the control siRNA group; ##*P*<0.01 vs. the insulin+control siRNA group.

### Measurement of ROS

DCF-DA was used to measure ROS as described [16]. Growth-arrested HMVECs were stimulated with 100 nM insulin for 5 minutes. After stimulation, HMVECs were incubated in phosphate buffered saline (PBS) containing DCF-DA (5 µM) for 15 minutes in a light-protected humidified chamber at 37°C. Cells were then rinsed in PBS and fixed with 10% buffered formalin. Images were obtained with a LSM 510 laser scanning confocal microscope (Carl Zeiss Inc., Thornwood, NY). The excitation was at 488 nm with an emission at 540 nm. The fluorescence image was collected by a single rapid scan with identical parameters for all samples. Fluorescent levels were expressed as percent increase over the control. The assays were carried out in duplicates, and data were obtained from three independent experiments.

**Figure 4:**
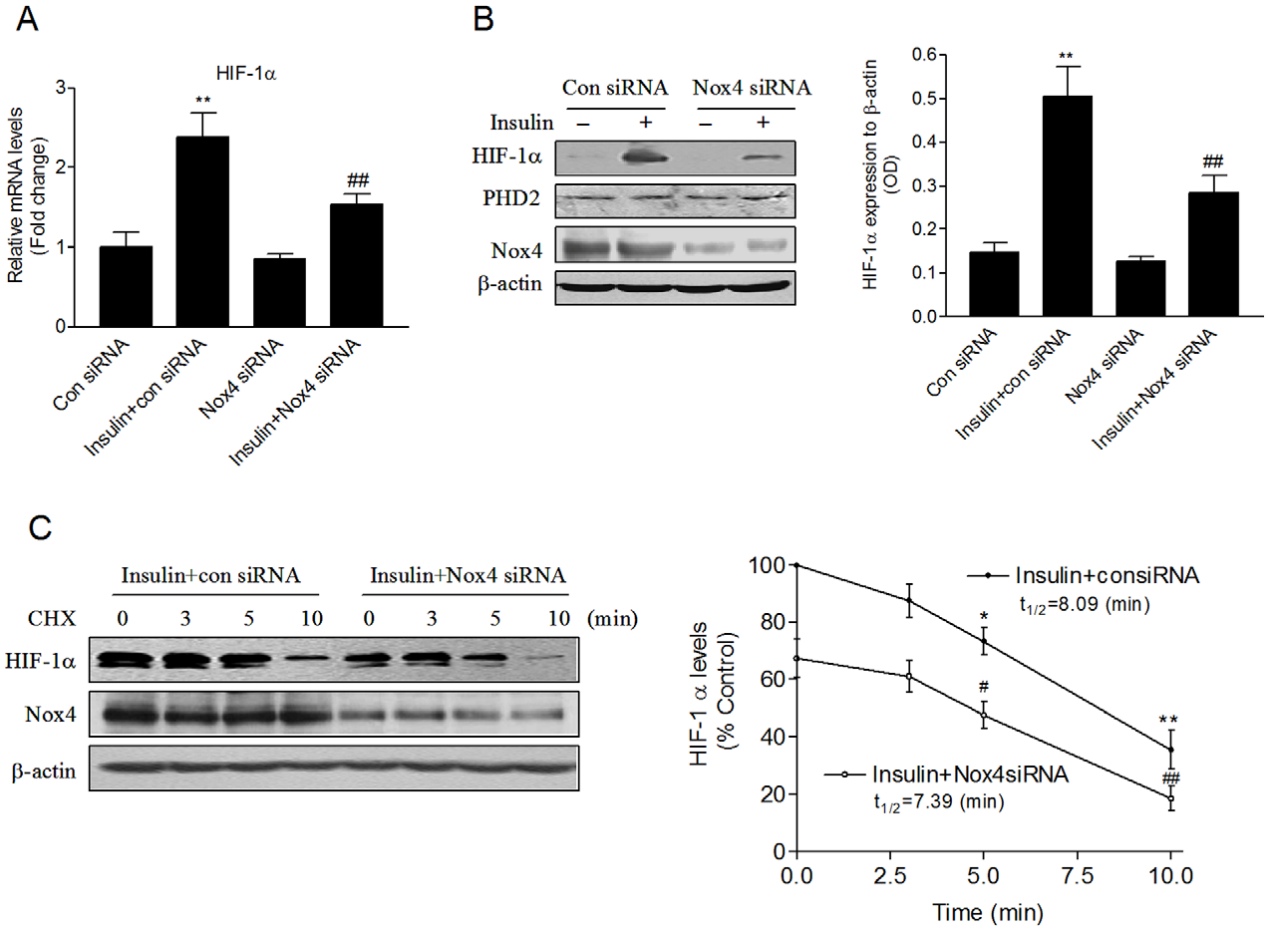
Nox4 mediates insulin-induced HIF-1α mRNA and protein expression. HMVECs were transfected with Nox4 siRNA or the control siRNA for 24 hours. The cells were growth arrested and then treated with 100 nM insulin for 12 hours. (A) HIF-1α mRNA levels were analyzed by real time-PCR. (B) HIF-1α, PHD2, Nox4, and β-actin protein levels were determined by immunoblotting (*P<0.05, **P<0.01 vs. the control siRNA group; #*P*<0.05, ##*P*<0.01 vs. the insulin+control siRNA group). (C) Cycloheximide (CHX) was added to a final concentration of 50 µg/ml, and the cells were harvested after being incubated for the indicated time in the presence of CHX and insulin. HIF-1α, Nox4, and β-actin protein levels were determined by immunoblotting (*P<0.05, **P<0.01 vs. zero-time control in insulin+con siRNA group; #*P*<0.05, ##*P*<0.01 vs. zero-time control in insulin+Nox4 siRNA group).

**Figure 5:**
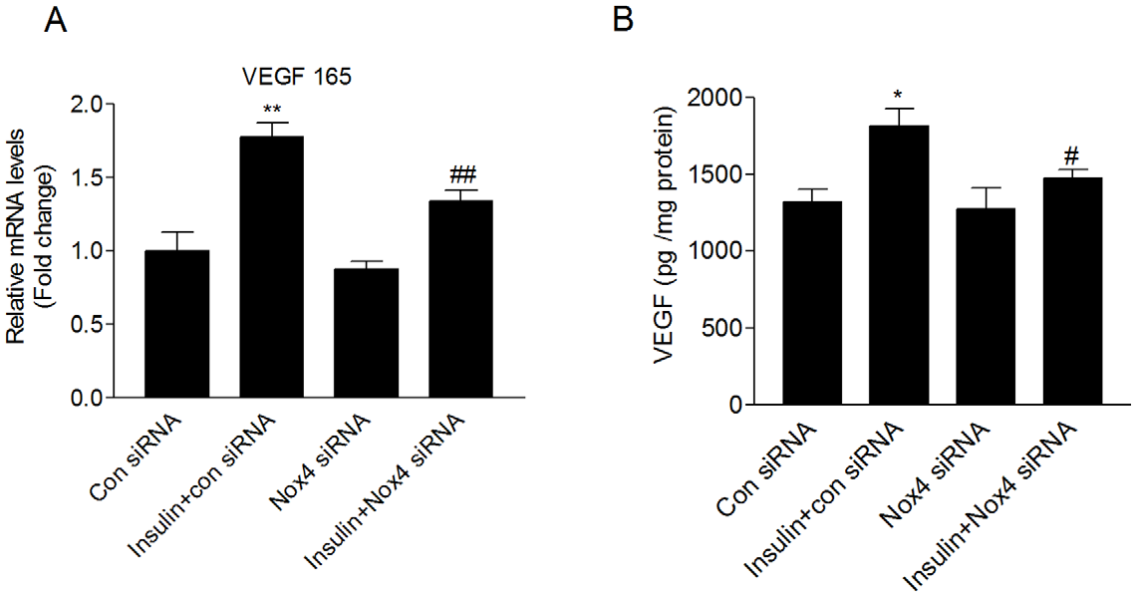
Nox4 mediates insulin-induced VEGF mRNA and protein expression. HMVECs were transfected with Nox4 siRNA or the control siRNA for 24 h. The cells were growth arrested and then treated with 100 nM insulin. (A) In 12 hours, VEGF 165 mRNA level was analyzed by real time-PCR. (B) In 24 hours, the VEGF protein levels in the supernatants were determined by ELISA assay. *P<0.05, **P<0.01 vs. the control siRNA group; #*P*<0.05, ##*P*<0.01 vs. the insulin+control siRNA group.

### Small interfering RNA (siRNA) transfection

Non-silencing (control) siRNA (100 nM) or Nox4 siRNA (100 nM) was transfected into HMVECs using the RNAi MAX transfection reagent according to the manufacturer's instructions (Invitrogen). The non-silencing siRNA was used as a negative control. 48 hours after transfection, the cells were starved for 16 hours followed by treatment with or without insulin.

**Figure 6:**
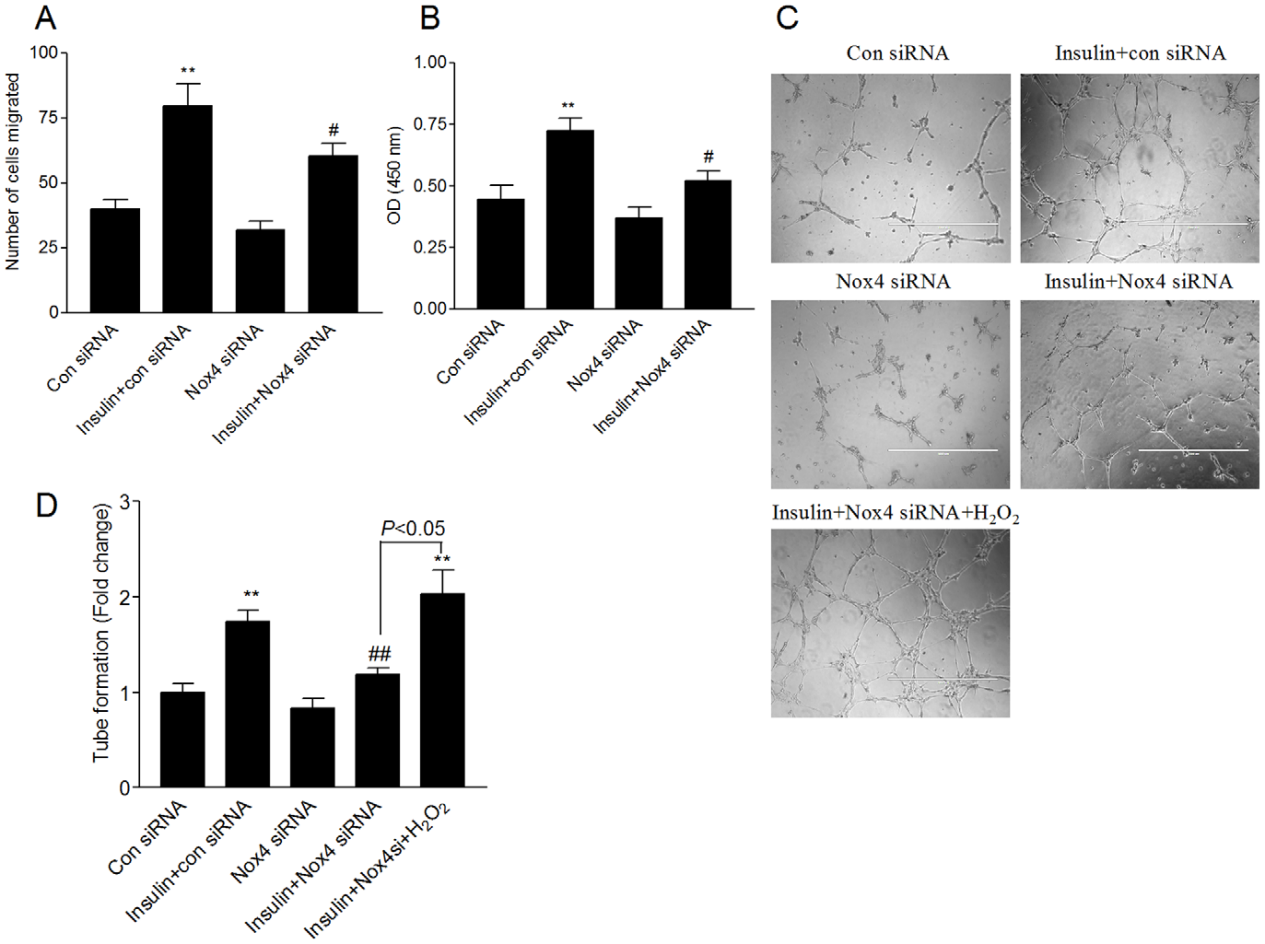
Nox4 mediates insulin-induced HMVEC migration, proliferation, and angiogenesis *in vitro*. HMVECs were transfected with the control or Nox4 siRNA for 24 hours. (A) The cells were growth arrested followed by stimulation with insulin (100 nM) for 10 hours. Cell migration was measured as described in Methods. The data are expressed as the fold change relative to the control. (B) Growth-arrested cells were treated with insulin (100 nM) for 24 hours. Cell proliferation was measured using CCK-8 assay as described in Methods. (C) and (D) HMVECs transfected with the control or Nox4 siRNA were seeded onto Matrigel and incubated with or without insulin (100 nM), treated or not with H_2_O_2_ (0.1 µM) for 12 hours. The relative tube lengths were normalized by the negative control. Bar = 1000 µm. **P<0.01 vs. the control siRNA group; #*P*<0.05, ##*P*<0.01 vs. the insulin+control siRNA group.

### Immunoblotting

Immunoblotting was performed as described previously [15].

### Transient transfection and luciferase assay

Transient transfection and luciferase assay were performed as described previously [23]. A 2.65 kb human VEGF gene promoter in pGL2-basic luciferase reporter and a 46 bp human VEGF gene promoter containing the HIF-1 binding site in pGL2-basic luciferase reporter were kindly provided by Dr. Jiang (Thomas Jefferson University, USA). The human wild-type Nox4 cDNA was cloned into the pcDNA3.1 vector. HMVECs were seeded in 12-well plates and cultured to 60–70% confluence. The cells were transiently transfected with VEGF reporter or pGL2-basic luciferase reporter and pCMV-β-galactosidase plasmid using Lipofectamine 2000 from Invitrogen (Carlsbad, CA). The transfected cells were cultured for 18–20 hours, and then incubated in serum-free medium for 12 hours followed by the incubation with insulin (100 nM) for 20 hours. Cells were lysed with reporter lysis buffer. Luciferase (Luc) activities of the cell extracts were determined using the Luciferase Assay System from Promega (Madison, WI). β-Galactosidase (β-gal) activity was measured. Relative Luc activity was calculated as the ratio of Luc/β-gal activity, and expressed as the ratio of VEGF/pGL2-basic. All luciferase assays reported here represent at least three independent experiments, each consisting of three wells per transfection.

### Reverse transcription-PCR (RT-PCR) and real-time PCR

Total cellular RNA was isolated from HMVECs using Trizol reagent (Invitrogen, Carlsbad, CA). 2 µg of RNA was processed directly to cDNA with Moloney Murine Leukemia Virus (M-MLV) reverse transcriptase (Promega, Madison, WI). The primers for human Nox4 were: 5′-TGTTGGATGACTGGAAACCA-3′ (forward), and 5′-TGGGTCCACAACAGAAAACA-3′ (reverse). The primers for human Nox1 were: 5′-GCCGACAAATACTACTACAC-3′ (forward), and 5′-GGACAGCAGATTGCGACA-3′ (reverse). The primers for human Nox2 were: 5′-AAGATAGCGGTTGATGGG-3′ (forward), and 5′-TGAGAATGGATGCGAAGG-3′ (reverse). The primers for human Nox3 were: 5′- CCTGGAAACACGGATGAG-3′ (forward), and 5′- CAGAAGAACACGCCAATAC-3′ (reverse). The primers for human Nox5 were: 5′- ATTGCTCGCTATGAGTGGC-3′ (forward), and 5′-CCCTTGGACGACCTTTGA-3′ (reverse). The primers for human VEGF 165 were: 5′-ATCTTCAAGCCATCCTGTGTGC-3′ (forward), and 5′-CAAGGCCCACAGGGATTTTC-3′ (reverse). The primers for human HIF-1α were: 5′-CTCAAAGTCGGACAGCCTCA-3′ (forward), and 5′-CCCTGCAGTAGGTTTCTGCT-3′ (reverse). The primers for human GAPDH are: 5′- CCACCCATGGCAAATTCCATGGCA-3′ (forward), and 5′- TCTAGACGGCAGGTCAGGTCCACC-3′ (reverse). Real-time PCR were performed according to the methods described previously [16].

### Quantification of VEGF protein

Human VEGF-A was quantified in the cell culture supernatant using ELISA assay kit from R&D Systems, Inc. (Minneapolis, MN). In brief, HMVECs were transfected with 100 nM Nox4 siRNA or control siRNA for 24 hours, the cells were growth arrested and then treated with insulin (100 nM) for 24 hours. The supernatants were collected, and total cellular protein was measured by protein assay kit (Bio-Rad Laboratories, Hercules, CA). VEGF-A production was expressed as pg/mg protein. The assays were carried out in duplicates, represent three independent experiments.

### Cell migration assay

Migration of HMVECs was determined by using transwell chambers (24-well plates, 8-μm pore size, Corning, Inc.) as described previously [24]. The top well was loaded with 1×10^5^ cells and insulin of 100 nM was added in the lower chamber to induce chemotaxis. After 10 hours of incubation, the cells were fixed and stained with 0.1% crystal violet. The number of cells migrated to the bottom side was counted under a light microscope. The assays were carried out in triplicates, represent three independent experiments.

### Cell proliferation assay

Cell proliferation was measured using a CCK-8 assay kit (Dojindo, Japan) as described previously [25]. HMVECs were transfected with the control or Nox4 siRNA for 24 hours, and growth arrested for 16 hours. The cells were then seeded in 96-well plate with insulin (100 nM) treatment for 24 hours. CCK-8 solution (150 µl) was added to each well followed by incubation for 2 hours. After incubation, the absorbance was measured at 450 nm using a microplate reader. The assays were measured in sextuplicates, represent three independent experiments.

### *In vitro* angiogenesis assay

*In vitro* endothelial cell tube formation assay in three dimensional Matrigel culture was used as a surrogate assay for angiogenic potential. 200 μl of growth factor-reduced Matrigel was preplaced in a 48-well plate. HMVECs were preincubated with serum-free medium for 16 hours. The cells (1.5×10^4^) treated with insulin (100 nM) were transferred onto 48-well Matrigel. Endothelial tube formation was evaluated after incubation for 12 hours. The capillary-like tube structures were examined using an inverted phase contrast microscope, and the tube formation index was determined by measuring the average length of tubes in five random fields from each well using an Axiovision software. The assays were carried out in triplicates, represent three independent experiments.

### Statistical analysis

Data are expressed as mean±SE from three independent experiments. Differences among groups were determined using prism software with one-way analysis of variance followed by the Newman-Keuls test, differences between two groups were assayed by two-tailed student *t*-test. The 0.05 level of probability was used as the criterion of significance.

## Results

### Insulin increases ROS production through a Nox4-based NADPH oxidase

We measured ROS generation using DCF-DA, a membrane-permeable dye that can be oxidized by intracellular ROS to the fluorescent product 2′,7′-dichlorodihydrofluorescein (DCF). The exposure of HMVECs to insulin (100 nM) for 5 minutes increased intracellular ROS generation, the flavoenzyme inhibitor diphenylene iodonium (DPI) inhibited insulin-derived ROS generation (Fig. 1A). Quantitative RT-PCR was performed to identify the Nox isoforms of NADPH oxidase in HMVECs. Nox4 was highly expressed in HMVECs, and the expression of Nox4 markedly exceeded that of Nox2 (Fig. 1B), which is consistent with the previous report [26]. Nox1 expression was detected at very low level only (ct value of 37). Nox3 and Nox5 were undetectable in HMVECs.

We next determined the role of Nox isoforms in insulin-stimulated ROS production. None of Nox isoforms protein expressions were changed by exposure to insulin for 5 minutes or 2 days (data not shown). Considering the predominant expressions of Nox4 and Nox2 in HMVECs, we used specific siRNA-mediated gene silencing to modulate Nox4 or Nox2 expression. Transfection of Nox4 siRNA resulted in a 50% decrease of Nox4 protein expression in HMVECs without decreasing Nox2 expression (Fig. 1C). ROS generation by insulin was significantly inhibited by Nox4 siRNA, whereas Nox2 siRNA had no significant effect on insulin-induced ROS production (Fig. 1D). These results show that insulin increases ROS production via a Nox4-based NADPH oxidase in HMVECs.

### Nox4 is involved in insulin-stimulated insulin receptor and IRS tyrosine phosphorylation, ERK1/2, and AKT activation

To explore the role of Nox4 in the insulin signaling pathway in HMVECs, we measured tyrosine phosphorylation of the insulin receptor and IRS-1. Insulin stimulated an increase in the tyrosine phosphorylation of insulin receptor and IRS-1 in cells transfected with the control siRNA. In cells transfected with the Nox4-specific siRNA, however, insulin-stimulated insulin receptor β tyrosine phosphorylation was decreased by 35% (*P<*0.01), and IRS-1 tyrosine phosphorylation was decreased by 32% (*P<*0.05) compared to the control response (Fig. 2A and 2B). Importantly, downregulation of Nox4 expression did not decrease the serine phosphorylation of IRS-1 induced by insulin (Fig. 2B). We next assessed the role of Nox4 in insulin-stimulated ERK1/2 and AKT activation. As shown in Fig. 2C and 2D, insulin increased the phosphorylation of ERK1/2 and AKT in cells transfected with the control siRNA, whereas the activation of ERK1/2 and AKT by insulin was significantly inhibited by Nox4 siRNA (*P<*0.05). As the MAPK phosphatase-1 (MKP-1) mediates the dephosphorylation and inactivation of ERK1/2 [27] and SHIP-1 (Src homology 2 domain–containing inositol 5′-phosphatase 1) negatively regulates the activation of AKT [28], we tested the effect of Nox4 silencing on MKP-1 and SHIP-1 expression. Importantly, downregulation of Nox4 had little effect on the expression of these phosphatases including MKP-1 and SHIP-1 (Fig. 2E).

### Nox4 mediates insulin-induced VEGF transcriptional activation through HIF-1

We investigated the potential involvement of Nox4 in the transcriptional activation of VEGF induced by insulin in HMVECs. HMVECs were transfected with a VEGF promoter reporter containing a 2.65-kb human VEGF promoter. Inhibition of ROS production by DPI or Nox4 siRNA inhibited insulin-induced VEGF transcriptional activation (*P<*0.01, Fig. 3A and 3B). To test whether the HIF-1 binding site at the VEGF promoter is important for Nox4-mediated VEGF transcriptional activation, a VEGF reporter containing a functional promoter fragment with the HIF-1 binding site was used. Inhibition of Nox4 expression also inhibited this VEGF reporter activity induced by insulin (*P<*0.01, Fig. 3C), indicating that Nox4 mediates insulin-induced VEGF transcription through HIF-1 activation. In addition, overexpression of wild-type Nox4 enhanced the VEGF reporter activity (Fig. 3D). Compared to cells transfected with the control vector, insulin-stimulated VEGF transcriptional activation was increased by 115% (*P<*0.01) in cells overexpressing wild-type Nox4 (Fig. 3D).

### Nox4 mediates insulin-induced HIF-1α and VEGF mRNA and protein expression

To determine whether Nox4 is involved in HIF-1α and VEGF expression, the mRNA and protein levels of HIF-1α and VEGF were measured in HMVECs transfected with Nox4-specific or control siRNA. Insulin increased the mRNA and protein levels of HIF-1α, which were inhibited by Nox4 siRNA (Fig. 4A and 4B). To examine the molecular mechanism of HIF-1α protein inhibition by Nox4 siRNA, we next investigated whether silencing Nox4 alters the stability of HIF-1α. Cycloheximide (CHX), a general inhibitor of protein synthesis, was used to prevent HIF-1α de novo synthesis. HMVECs treated with CHX in combination with insulin displayed a time-dependent decrease of HIF-1α protein level (Fig. 4C). Knockdown of Nox4 expression did not significantly change the rate of HIF-1α degradation (Fig. 4C). Moreover, downregulation of Nox4 had little effect on HIF prolyl hydroxylase domin 2 (PHD2) protein expression (Fig. 4B). Similarly, Inhibition of Nox4 expression also inhibited insulin-induced VEGF mRNA and protein expression (Fig. 5A and 5B).

### Nox4 mediates insulin-induced HMVEC migration, proliferation and angiogenesis *in vitro*

To examine the role of Nox4 in insulin-induced HMVEC migration, proliferation and angiogenesis *in vitro*, we knocked down Nox4 using siRNA in HMVECs. We observed that inhibition of Nox4 expression decreased insulin-induced cell migration (*P<*0.05, Fig. 6A) and cell proliferation (*P<*0.05, Fig. 6B). The functional effect of Nox4 on angiogenic potential was tested by three dimensional Matrigel tube forming assays. The results revealed that knockdown of Nox4 expression resulted in significant inhibition of insulin-induced tube formation (*P<*0.01, Fig. 6C and 6D). Of note, compromise in Matrigel tube formation of Nox4 knockdown could be significantly corrected by administration of exogenous H_2_O_2_ to cells (*P<*0.05, Fig. 6C and 6D).

## Discussion

Previous studies have shown that ROS are involved in insulin signaling pathways [3], [7]. However, the role of Nox4 in insulin-stimulated HIF-1α and VEGF expression, and angiogenic responses remains unknown. In this study, we provided evidence that Nox4-derived ROS have an important role in activating ERK1/2 and AKT, modulating HIF-1α-dependant VEGF expression, and angiogenic responses induced by insulin.

In this study, we showed that insulin-induced ROS production was reduced by the flavoenzyme inhibitor DPI or Nox4 siRNA, but it was not affected by Nox2 siRNA (Fig. 1). These data suggest that Nox4 is involved in the generation of ROS by insulin in HMVECs. Similar findings were reported previously for insulin in adipocytes [4], [29]. Indeed, Nox4 is constitutively active and ROS formation by Nox4 is linked to its expression [30]–[31]. We found that a short (e.g. 5 minutes) exposure to insulin had no effect on the expression of Nox4 or other Nox isoforms (data not shown), but it enhanced ROS generation. It was known that Nox4/p22phox complex formation is required for Nox4-derived ROS [30]. Recent study has demonstrated that IGF-I stimulated Nox4/p22phox complex formation within 5 minutes [32]. The exact mechanism of acute insulin-induced ROS production still needs to be identified.

We found that knockdown of Nox4 expression reduced the tyrosine phosphorylation of IR-β and IRS-1, but did not change the serine phosphorylation of IRS-1 in HMVECs (Fig. 2), suggesting that insulin-stimulated tyrosine phosphorylation of insulin receptor and its major receptor substrate is regulated by Nox4. These findings are consistent with the previous observation by Biswas and colleagues, which demonstrated that insulin-induced Nox4-linked generation of ROS activated insulin receptor tyrosine kinase by inhibiting protein-tyrosine phosphatase 1B (PTP1B) in adipocytes [4]. Moreover, we found that inhibition of Nox4 had little effect on the expression of the phosphatases such as MKP-1 and SHIP. Our results are in contrast to that study in human preadipocytes, which demonstrated that downregulation of Nox4 attenuated MKP-1 expression, and promoted ERK1/2 activation and cell proliferation in response to insulin [33]. Differences between our findings and that study may be due to the differences in the cells used. Furthermore, we found that Nox4 gene silencing had a much greater inhibitory effect on insulin-induced AKT activation than ERK1/2 activation (Fig. 2C and 2D). It has been shown that PI3K/AKT pathway is essential for retinal angiogenesis in the zebrafish [34]. Thus, our data suggest that Nox4 plays a more important role in the PI3K/AKT pathway than in the MAPK pathway for insulin signaling.

In this study, knockdown of Nox4 expression inhibited the transcriptional activity of VEGF reporter containing the HIF-1 binding site, indicating that Nox4 mediates insulin-induced VEGF transcription activation through HIF-1. Overexpression of Nox4 alone was sufficient to increase VEGF transcriptional activity, and Nox4 enhanced insulin-stimulated the activation of VEGF (Fig. 3). In addition, we also found that insulin-induced HIF-1α and VEGF expression were blocked by PI3-kinase inhibitor LY294002 and MEK1 inhibitor U0126 (data not shown). Thus, our findings suggest that Nox4-derived ROS regulate insulin-induced HIF-1α and VEGF expression through PI3K/AKT and ERK1/2 pathways.

Our findings demonstrated that insulin stimulated HIF-1 α mRNA and protein expression. In deed, consistent with our observation, it was recently demonstrated that insulin induced HIF-1 α expression in mRNA and protein level in adipocytes [35]. Here, we showed that inhibition of Nox4 impaired insulin-stimulated mRNA and protein expression of HIF-1α (Fig. 4A an 4B), suggesting that Nox4-derived ROS are required by insulin in the stimulation of HIF-1α mRNA and protein expression. Moreover, we found that knockdown of Nox4 expression did not change the expression of PHD2 and the rate of HIF-1 α degradation (Fig. 4B and 4C), indicating that HIF-1 α repression by silencing Nox4 is not through the decrease of stabilization of HIF-1 α protein. This is in line with the previous observation in 786-O renal carcinoma cells, which demonstrated that knockdown of Nox4 by siRNA decreased HIF-2 α mRNA and protein levels independent of pVHL, suggesting that Nox4-derived ROS regulate HIF-2 α by a translational mechanism [36]. Similarly, in the present study, Nox4 regulates the PI3K/AKT signaling, which is important for the translation of HIF-1α protein expression. Thus, it is likely that Nox4 mediates insulin-stimulated HIF-1 α expression through a transcriptional and translational mechanism in HMVECs. In the present study, we have demonstrated that Nox4-derived ROS are essential for HIF-1α-dependent VEGF expression induced by insulin. In contrast to our findings, a previous study showed that Nox3-derived ROS regulated insulin-induced VEGF through the activation of Sp1, but not by HIF-1α in HepG2 cells [37]. It suggests that insulin-stimulated ROS modulate VEGF expression through multiple pathways, depending on different Nox isoform involved.

We found that inhibition of Nox4 impaired insulin-stimulated cell migration, cell proliferation, and tube formation in HMVECs, and the impaired tube formation by knockdown of Nox4 can be corrected by exogenous H2O2. These results support an involvement of Nox4-derived ROS in insulin-induced angiogenesis *in vitro*. In fact, Nox4 has been associated with angiogenesis, overexpression of Nox4 was sufficient to promote endothelial proliferation, migration, and tube formation [6]. A recent study has shown that Nox4 (−/−) mice exhibited attenuated angiogenesis, further confirming the important role of Nox4 in angiogenesis [38].

In summary, the data presented here demonstrated that Nox4 is a critical mediator of insulin-mediated ROS generation, HIF-1α and VEGF expression, endothelial cell migration, and angiogenesis *in vitro*. Nox4 may be an attractive therapeutic target for diabetic retinopathy caused by intensive insulin treatment.

## Supporting Information

Table S1 Summary of antibodies.
(DOC)
