# Supplementary material for: NADPH Oxidase 4 Mediates Insulin-Stimulated HIF-1α and VEGF Expression, and Angiogenesis In Vitro
Source: PLoS One. 2012 Oct 29;7(10):e48393. doi: 10.1371/journal.pone.0048393 (PMC3483150; doi:10.1371/journal.pone.0048393)
Supplement: Table S1 — Summary of antibodies. (DOC) [file pone.0048393.s001.doc]

**Table S1**

| Name | Catalogue number | Dilution | Company |
| --- | --- | --- | --- |
| Nox4 | SC21860 | 1:500 | Santa Cruz |
| Nox2 | SC130543 | 1:500 | Santa Cruz |
| p-insulin Rβ | SC25103 | 1:1000 | Santa Cruz |
| insulin Rβ | SC711 | 1:1000 | Santa Cruz |
| p-IRS-1(Try1229) | SC17202 | 1:1000 | Santa Cruz |
| IRS-1 | SC559 | 1:1000 | Santa Cruz |
| p-ERK1/2 | SC7383 | 1:1000 | Santa Cruz |
| ERK2 | SC154 | 1:1000 | Santa Cruz |
| PHD2 | SC34920 | 1:500 | Santa Cruz |
| MKP-1 | SC370 | 1:1000 | Santa Cruz |
| SHIP-1 | SC8425 | 1:1000 | Santa Cruz |
| p-AKT | CST4051 | 1:1000 | Cell Signaling |
| AKT | CST9272 | 1:1000 | Cell Signaling |
| p-IRS-1(Ser 612) | CST3193 | 1:500 | Cell Signaling |
| HIF-1α | BD610959 | 1:500 | BD Pharmingen |
| β-actin | A5316 | 1:5000 | Sigma |

**Table S1. Summary of antibodies.**
